# Protocol to study the contribution of biomechanics in osteoarthritis development using a cartilage-on-chip model

**DOI:** 10.1016/j.xpro.2026.104365

**Published:** 2026-02-11

**Authors:** Elodie Faure, Veronique Chobaz, Diego de Haro, Elsa Lauwers, Nathalie Busso, Carlo Alberto Paggi, Sonia Nasi

**Affiliations:** 1 Division of Rheumatology, Department of Musculoskeletal Medicine, Lausanne University Hospital, 1010 Lausanne, Switzerland; 2 Division of Infectious Diseases, Department of Medicine, Lausanne University Hospital, 1010 Lausanne, Switzerland; 3 Chrn on-chip Biotechnologies B.V. (aka chiron), Maastricht 6229 GS, the Netherlands

**Keywords:** Biotechnology and bioengineering, Cell Biology, Molecular Biology

## Abstract

Here, we present a protocol to study biomechanics and calcification in osteoarthritis using a cartilage-on-chip model. We describe steps for culturing human chondrocytes in 3D hydrogels and subjecting them to physiological loading. We then detail procedures for assessing spontaneous calcification via alizarin red S staining, gene expression using qPCR, protein expression by immunofluorescence, and protein secretome analysis through the Olink proximity extension assay. This protocol is adaptable and allows exploration of cartilage degeneration mechanisms under controlled conditions.

## Highlights

• Procedure for assessing effects of mechanical loading in a 3D cartilage-on-chip system
• Steps for imaging pathologic calcification of chondrocytes
• Procedures to analyze gene expression by RT-qPCR and protein levels by Olink

## Abstract (editor highlights)

Publisher’s note: Undertaking any experimental protocol requires adherence to local institutional guidelines for laboratory safety and ethics.

## Graphical abstract

**Figure:**
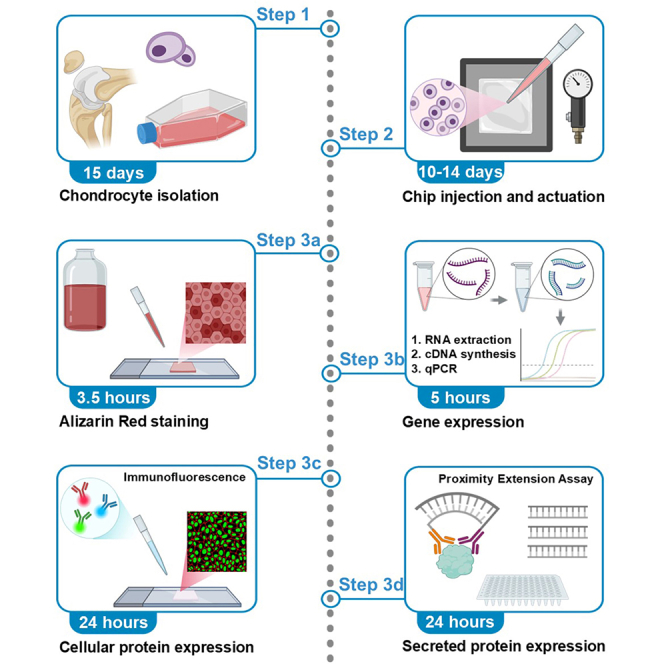

## Before you begin

Osteoarthritis (OA) is a joint degenerative disease affecting, 528 million people worldwide in 2019. It causes joint pain, swelling, stiffness and progressively disrupts tissues, leading to a slow and steady worsening of the condition. OA is most commonly seen in knees, hips, spine and hands. Multiple parameters can contribute to the initiation and progression of the disease, including hereditary factors, gender, mechanical overload and intra articular calcium deposition. Articular cartilage calcification - the abnormal deposition of calcium phosphate crystals within the cartilage matrix - is increasingly being considered as one of the critical drivers of OA. So far, very little is known about the mechanistic effects leading to calcification, as the parameters to consider include both phosphate and the mechanical stimuli within the joint. To assess these critical OA parameters, we have developed a cartilage-on-chip model using human chondrocytes donor. Here, the model was able to recapitulate physiological active mechanical stimuli onto a 3D construct containing human articular chondrocytes.

In this protocol, we present the first step-by-step guide to culture human articular cartilage within a 3D hydrogel construct under physiological condition using a commercially available cartilage-on-chip system. Here, we provide a complete workflow to analyze the effects of biomechanics on spontaneous calcification, gene expression and protein production.

This protocol will outline how to operate the cartilage-on-chip devices with one mechanical stimulation pattern. Additionally, the protocol will describe how to perform cell calcification staining using Alizarin Red S, gene expression by RT-qPCR as well as protein production analysis using two methods: multiple immunofluorescence staining for markers expected to be robustly expressed under our experimental conditions (TRVP4, LOX and *β*-actin) and targeted proteomic analysis using Olink’s Proximity Extension Assay (PEA).

While tailored for primary chondrocytes and specific amounts of cells, this platform can easily be adapted to immortalized cell lines and higher cell concentrations. Each of the processes or analytical techniques used in this protocol will be described step-by-step, and diverging from the suggested method may lead to different outputs.

### Innovation

The cartilage-on-chip model is the first of its kind organ-on-chip platform, allowing for active compressive mechanical stimulation forces (from 0 to 1000mbar) onto a 3D cell-hydrogel structure. Moreover, it is the first system applying lateral - rather than vertical - compression, allowing for both endpoint imaging (after cell-fixation) and real-time visualization of cells during mechanical stimulation. The monolithic device is comprised of a mechanical actuation, a 3D cell-hydrogel chamber and a perfusion channel to provide nutrients to the system. The mechanical stimulation is applied via a regulated pressure pump and is applied by deforming an elastic membrane. The design has been created to simulate both healthy (<20%) and diseased (>20%) cartilage forces. The platform allows for various frequencies (0.01 to 2 Hz at 300 mbar input) or type of force input (0 to 1000 mbar), providing a wide range of flexibility to the user. This platform has been created to simulate the key mechanical stimulation characteristics present on the knee cartilage.

### Institutional permissions

Human chondrocytes were isolated from osteoarthritic knees of patients undergoing knee replacement surgery. The collection and use of human samples were approved by the Centre Hospitalier Universitaire Vaudois ethical committee and patient written informed consent was obtained.

## Key resources table

**Table:** 

REAGENT or RESOURCE	SOURCE	IDENTIFIER
**Antibodies**		

TRPV4 Antibody [Alexa Fluor 594, dilution 1/150]	Novus Biologicals	NBP2-41262AF594
LOX Polyclonal antibody [dilution 1/200]	Proteintech	17958-1-AP
β-Actin Antibody [Alexa Fluor 647, dilution 1/150]	Novus Biologicals	NB600-503AF647
AffiniPure Goat Anti-Rabbit IgG (H+L) [Alexa Fluor 488, dilution 1/200]	Jackson ImmunoResearch	111-545-003

**Biological samples**		

Human knee chondrocytes (from osteoarthritic patients)	Lausanne University Hospital	N/A

**Chemicals, peptides and recombinant proteins**		

DMEM, high glucose, GlutaMAX Supplement	ThermoFisher	61965026
Fetal Bovine Serum (FBS) Supreme	Pan Biotech	P30-3031
Penicillin-Streptomycin	Sigma-Aldrich	P0781
Trypsin-EDTA PBS 1:250 (0.05%/0.02%) w/o Ca/Mg	BioConcept	5-51F00-H
Liberase TM Research Grade	Roche	5401127001
Phosphate-buffered saline (PBS)	Bischel Interlaken	100 0 324
Alizarin Red S	Sigma-Aldrich	A5533-25G
UltraPure Low Melting Point Agarose	Invitrogen	16520050
Ammonium hydroxide solution (NH_4_OH)	Sigma-Aldrich	221228
Methanol	Sigma-Aldrich	34860
Albumin (BSA) Fraction V (pH 7.0)	Sigma-Aldrich	A1391
Paraformaldehyde 16% Aqueous Solution EM Grade	Electron Microscopy Sciences	15710-S
Triton X-100	Sigma-Aldrich	T8787
Mounting Medium With DAPI - Aqueous, Fluoroshield	Abcam	ab104139

**Critical commercial assays**		

RNeasy Mini Kit	QIAGEN	74106
iScript cDNA synthesis kit	Bio-Rad	1708890
IQ SYBR green Mix	Bio-Rad	1708887
Olink Target 96 Inflammation kit	Olink	95302

**Oligonucleotides**		

Human GAPDH (forward and reverse 5′-3′)	GAT TTG GTC GTA TTG GGC GC	CTC GCT CCT GGA AGA TGG TG
Human LOX	GCA TAC AGG GCA GAT GTC AGA	TTG GCA TCA AGC AGG TCA TAG

**Software and algorithms**		

ImageJ/Fiji software	https://imagej.net/software/fiji/#downloads	

**Other**		

Optical microscope	Olympus BX43	Olympus
Optical microscope objectives	UplanFl 4/0.13 Japan	Olympus
	UplanFl 10/0.30 Japan	Olympus
Confocal microscope	Zeiss LSM 880 with Airyscan	Zeiss
Confocal microscope objectives	EC Plan Neofluar 20×/0.5 NA Ph2 DIC II	Zeiss
	Plan Neofluar 63×/1.25 NA oil immersion DIC III	Zeiss
Nanodrop	NanoDrop One	Thermo Fisher Scientific
Thermocycler block	C1000 Thermal Cycler	BioRad
qPCR machine	CFX Opus 96 Real-Time PCR System	Biorad
Qualitx Regular tips 200ul	Socorex	3070.0200B
Tissue Culture Flasks 150	TPP	90151
Eppendorf Safe-Lock Tubes (1.5mL)	Eppendorf	0030120086
Hard-Shell PCR Plate 96-well	Bio-Rad	HSP9601
Microseal B Adhesive Seals	Bio-Rad	MSB1001B
Fisherbrand Grade 600 Cellulose General Purpose Filter Paper: Discs	Fisher Scientific	11852723
Kimtech Science Precision Wipes	Kimberly-Clark	05511
SuperFrost Plus Adhesion slides, White (75mm x 25mm)	Epredia	J1800AMNZ
Glass bottle	Duran flask	
Swann-Morton Carbon Steel Sterile Scalpel Blades N°23	Swann-Morton	0210
70 μm Cell Strainer	Falcon	352350

## Materials and equipment

**Table:** Normal Medium

Reagent	Final concentration	Amount
DMEM	89%	445 mL
FBS	10%	50 mL
Penicillin-Streptomycin	1%	5 mL
Total	100%	500 mL

• Store the solution at 4°C form maximum 1 month.
• Before use, mix the solution well and place it at 37°C.

**Table:** Alizarin Red S Solution

Reagent	Final concentration	Amount
Alizarin Red S	0.5%	0.5 mL
Demineralized H_2_O	99.5%	99.5 mL
NH_4_OH	To adjust pH to 4.2	
Total	100%	100 mL

• Filter the solution with a paper filter.
• Store the solution at 4°C form maximum 1 month.
• Before use, mix the solution well and place it at 20°C.

## Step-by-step method details

### Human chondrocyte isolation and preparation

**Timing: 15 days**

**Timing: 24 h (for step 1)**

**Timing: 14 days (for step 2)**

**Timing: 30 min (for step 3)**

This section describes how to isolate, amplify and prepare primary human chondrocytes for injection in the devices.

1. Isolation.
  a. Isolate chondrocytes form osteoarthritic cartilage.^1^ Briefly, separate macroscopically chosen undamaged cartilage from the subchondral bone using a scalpel.
  b. Cut it in small pieces and wash twice with 37°C warm PBS and twice with DMEM.
  c. Digest cartilage for around 10 hours in 10% Liberase (Roche) DMEM.
  d. The following day, pass the digested tissue through a 70 μm filter to obtain articular chondrocytes.
2. Amplification of cells.
  a. Count the isolated cells and plate them at 1 × 10^6^ cells per T150 flask.
  b. Let them amplify in DMEM + 10% FBS for 14 days.
3. Preparation.
  a. Once cells reach confluency, wash with 10 mL of 37°C warm PBS twice.
  b. Detach cells with 5 mL 37°C warm Tris-EDTA.
  c. Adjust cell concentration to 6 × 10^6^ cells/mL in normal medium.

### Gel preparation, cell injection, and chip actuation

**Timing: 10–14 days**

**Timing: 30 min (for step 4)**

**Timing: 3 h (for step 5)**

**Timing: 10–14 days, 2 h related to the daily actuation of the devices (for step 6)**

This section describes how prepare the agarose gel, including injection of primary chondrocyte and biomechanical actuation.

**Figure 1:**
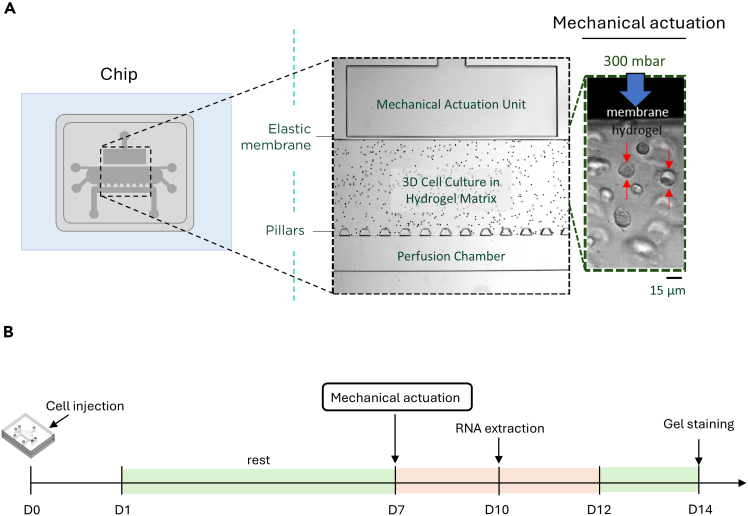
Cartilage-on-chip and schematic protocol (A) Cartilage-on-chip system. Cells are injected inside a chamber and subjected to 300 mbar loading. (B) Schematic protocol. After injection, cells rest in non-treated medium (NT, DMEM + 10% FBS). After 7 days, cells are subjected to mechanical loading (300 mbar) and cultured in non-treated medium (NT). RNA is extracted after 2 days of stimulation followed by 1 resting day, and gels are stained after 5 consecutive days of actuation followed by 2 resting days. Media is collected before actuation and before gel collection.

4. Gel preparation.
  a. Prepare a 4% agarose gel by heating 2 g of low melting agarose in 55 mL of sterile PBS in an Erlenmeyer.
    ***Note:*** Due to PBS evaporation during gel heating, dissolve the powder in 55 mL instead of 50 mL of PBS.
  b. Once powder clumps disappear, cover the Erlenmeyer with a sterile aluminum foil and place it in a 57°C bath for 10 min to remove bubbles.
5. Cell injection.
  ***Note:*** Injection of cells in the chip devices is described here^2^ and a schematic representation is provided in Figures 1A and 1B.
  a. Briefly, mix 400 μL of cell suspension with 400 μL of prepared gel on 37°C heating block.
  b. Pipette the cell-agarose suspension up and down until the two solutions are completely mixed.
  c. Withdraw 50 μL of cell-agarose suspension and gently inject it in the cell chamber until this appears filled.
6. Chip actuation.
  ***Note:*** After 7 days of culture in normal medium, begin the actuation step. Detailed instructions for chip actuation, including manipulation of the machine, connection and disconnection of the cartilage-on-chip from the actuation unit can be found here.^2^ A schematic representation is also shown in Figures 1A and 1B.
  a. Before each actuation, aspirate the culture medium from the top of the chip using a P200 pipette or an absorbent paper.
  b. Collect the medium from the perfusion channels using a P20 pipette set to 20 μL. Transfer the collected medium to a tube and store at −20°C for PEA analysis.
    ***Note:*** This tube will contain the cumulative medium from day 2 to the end of the experiment.
  c. Add approximately 150 μL of fresh culture medium inside the channel and to the top of the chip to avoid gel dehydration.
  d. Actuate 1 h at 300 mbar using 1 Hz frequency during:
    i. 2 consecutive days to study gene expression.
    ii. 5 consecutive days to study gel staining and proteomics.
      ***Note:*** On actuation days, perform the actuation at the same time each day.
  e. After the required time of actuation (2 or 5 days), let the chips rest for:
    i. 1 day to study gene expression.
    ii. 2 days to study gel staining and proteomics.
  f. Collect and store medium before harvesting the gel.
    ***Note:*** Detailed instructions for chip actuation, including manipulation and connection of the machine have been published here.^2^

### Gel staining with alizarin red

**Timing: 3 h 30 min**

This section describes how to stain gels with Alizarin Red staining, to evaluate calcification.

7. Alizarin Red Staining.
  a. Take out the agarose gel from the chip, place it on a microscope slide.
  b. Add 100 μL of PBS to the hydrogel to wash it.
  c. Repeat step b.
  d. Fix cells by adding 100 μL of frozen (−20°C) methanol.
  e. Place the slide presenting the extracted hydrogel at 4°C for 10 min.
  f. Repeat this step for each of the hydrogels being assessed.
  g. Wash the hydrogel with 100 μL of PBS.
  h. Wash the hydrogel with 100 μL of distilled water.
  i. Stain the hydrogel with 100 μL of 0.5% Alizarin Red (pH 4.2) and keep 1 h at 20°C.
  j. Wash the hydrogel with 100 μL of tap water for 30 sec.
  k. Repeat three times step j.
  l. Remove the tap water and replace with 100 μL of PBS to the hydrogel.
  m. Take pictures with an optical microscope Olympus BX43: picture the entire gel (with lens UplanFl ×4 magnification, 0.13 numerical aperture NA); 3 fields with 2 depths (with lens UplanFl ×10 magnification, 0.30 numerical aperture NA).
  n. Using Fiji software, quantify the red signal inside the cells and compare between the conditions.

### Gene expression analysis

**Timing: 5 h**

**Timing: 1 h 30 min (for step 8)**

**Timing: 1 h (for step 9)**

**Timing: 2 h 30 min (for step 10)**

This section describes how to obtain RNA and subsequent cDNA from cells embedded in the gels, to perform gene expression analysis via qPCR.

***Note:*** This section is divided into 3 parts that can be performed on 3 different days: sample RNA extraction, cDNA synthesis and qPCR.

8. RNA extraction.
  ***Note:*** RNA sample lysis has been reported here.^2^ Next, the RNA extraction of the samples is performed with Qiagen RNeasy Mini Kit (Qiagen, cat#74106) and finally, the RNA samples are assessed using the Nanodrop to determine purity of the samples.
  a. Aspirate the medium from the perfusion channel and wash with 30 μL PBS by pipetting up and down 10 times. Remove the PBS.
  b. Add 50 μL RNA dissociation buffer into the perfusion channel and pipette up and down for at least 20 times.
  c. Collect the RNA dissociation buffer.
    ***Optional:*** Protocol can be paused here. Chips can be kept at −80°C.
  d. Add 1 volume of 70% ethanol to the samples and mix well by pipetting.

Example: if the sample has a volume of 50 μL, add 50 μL of 70% ethanol.

  e. Transfer the entire volume (max 700 μL) to a RNeasy spin column placed in a 2 mL collection tube (as supplied per kit).
  f. Centrifuge for 15 sec at ≥ 8000 × *g*.
  g. Discard the flow-through.
  h. Add 700 μL Buffer RW1 (supplied per kit) to the RNeasy spin column.
  i. Centrifuge for 15 sec at ≥ 8000 × *g*.
  j. Discard the flow-through.
  k. Add 500 μL Buffer RPE (supplied per kit) to the RNeasy spin column.
  l. Centrifuge for 15 sec at ≥ 8000 × *g*.
  m. Discard the flow-through.
  n. Add 500 μL Buffer RPE (supplied per kit) to the RNeasy spin column.
  o. Centrifuge for 2 min at ≥ 8000 × *g*.
  p. Place the RNeasy spin column in a new 2 mL collection tube and discard the old collection tube with flow-through.
  q. Centrifuge for 1 min at 17000 × *g*.
  r. Place the RNeasy spin column in a new 1.5 mL collection tube.
  s. Add 30 μL of RNase-free water directly to the spin column membrane.
  t. Centrifuge for 1 min at ≥ 8 000 × *g*.
  u. Discard the spin columns, and place the collection tube on ice.
  v. Perform RNA quantification/qualification by measuring 1 μL of RNA sample using the Nanodrop.

9. cDNA synthesis.
  ***Note:*** The cDNA synthesis of the RNA samples is performed with BioRad iScript cDNA synthesis kit. This kit includes 5× reverse-transcription reaction mix, iScript reverse transcriptase and nuclease-free water.
  a. Calculate the volume of RNA to be used for each sample as per example below.

**Table:** 

Condition	Concentration
Sample X RNA concentration	20 ng/μL (fixed)
User-defined RNA final mount	X (calculated based on Nanodrop)
Volume of RNA (sample X)	X /20 ng/μL = X μL of RNA solution
  b. Calculate the volume of nuclease-free water to reach a final volume of 15 μL for each sample.

Example: nuclease-free water = 15 μL - 5 μL of RNA solution = 10 μL.

  c. Calculate the amounts for each of the samples.
  d. Track the results into a controlled excel file.
  e. Prepare a box of ice to place the Eppendorf tubes and RNA during the entire length of the process.
  f. Take the iScript cDNA synthesis kit with nuclease-free water, reverse transcriptase and iScript reverse-transcription reaction mix from the −20°C freezer and place them on ice.
  g. Take the RNA samples and place them on ice.
  h. Take the PCR strip and label the tubes as follow:

**Table:** 

Operator initials	ELW	(3 letters)
Date	06JUL25	(DD/MON/YR)
Sample reference code	TAR06JUN25NT	(project/date/type of sample
  i. Pipette the following components in the pre-labeled PCR strip.

**Table:** 

Material	Volume
5× iScript reaction mix	4 μL
iScript reverse transcriptase	1 μL
Nuclease-free water	x μL (as calculated above)
RNA template (100 fg to 1 μg total RNA)∗	x μL (as determined above)
Total volume	20 μL
    ***Note:*** When using larger amounts of input RNA (>1 μg), the reaction should be scaled up, e.g., 40 μL reaction for 2 μg, or 100 μL reaction for 5 μg to ensure optimum synthesis efficiency.
    ***Note:*** The total volume of RNA needed for each cDNA synthesis reaction depends on the amount of RNA we want to use and its concentration. Values between 100–500 ng are commonly used and recommended.
    ***Note:*** Do not use the same tips for different samples to avoid cross contamination.
  j. Once all the components are present, pipette up and down 10 times to allow appropriate mixing.
  k. Seal the PCR strips with their caps, spin down and run the samples in the thermocycler block using the following protocol:

**Table:** 

Parameters thermocycler
5 minutes at 25°C
20 minutes at 46°C
1 minute at 95°C
Hold at 4°C (optional)
    ***Optional:*** If you do not proceed to perform qPCR place the labeled PCR strips into the −20°C freezer.

10. qPCR analysis.
  ***Note:*** The range of cDNA per PCR reaction (well) is between 2–100 ng. However, 5–10 ng in 20 μL of qPCR reaction is generally used as it ensures a good amplification of your target genes.

For simplicity purposes this protocol considers the initiation of the qPCR at a different date than the cDNA synthesis.

  a. Calculate the total amount of cDNA required for each sample.
  b. Establish a final concentration of cDNA per volume (e.g., 5 ng/μL).
    ***Note:*** It is generally assumed that the RNA is transformed in cDNA in a 1:1 ratio or proportionally to the starting RNA concentration.
  c. Calculate the total amount of cDNA required to have 10 ng for each qPCR sample.
    ***Note:*** The final concentration of cDNA could vary as already stated. It is important to keep the same final concentration for each sample considered.

Example: cDNA per qPCR reaction = 10 ng/(5 ng/μL) = 2 μL.

  d. Calculate the total amount of nuclease-free water required for each qPCR sample.
    Example: nuclease-free water = 8 μL–2 μL cDNA per qPCR reaction = 6 μL.

**Table:** 

Material	Volume
IQ SYBR green Mix	10 μL
Reverse primer (10 μM)	1 μL
Forward primer (10 μM)	1 μL
cDNA sample	x μL (as calculated above)
nuclease-free water	x μL (as calculated above)
Total	20 μL
  e. Prepare a box of ice to place Eppendorf and cDNA during the entire length of the process.
  f. If not already diluted, dilute the 100 μM primer stock solution to 10 μM working solution in nuclease-free water.
  g. Pipette the appropriate amount of nuclease-free water based on the initial primer stock.
    ***Note:*** Depending on where you purchase the primers they may have different initial primer concentration.
  h. Thaw the cDNA samples and place them in the box of ice.
  i. Take a qPCR 96-well plate and place it on the box of ice.
  j. Prepare a MasterMix per primer with SYBR Green, Forward and Reverse primer and nuclease-free water. Keep this on ice and protect from light.
  k. First add the cDNA sample to the 96-well plate.
  l. Then, add the primer MasterMix to the 96-well plate.
    ***Note:*** Depending on the equipment used it is possible to use qPCR 384 wells plate.
    ***Note:*** For each sample there should be sufficient cDNA to have triplicates for each of the primers considered.
    ***Note:*** Add a negative control prepared during cDNA synthesis, and a water control (primer MasterMix without cDNA).
  m. Pipette up and down 10 times each sample to appropriately mix the solution.
  n. Seal the plate with the appropriate sealing adhesive.
  o. Spin down the plate with a 15 sec short spin in the centrifuge.
  p. Turn on the Biorad qPCR machine and select the applicable pre-existing qPCR protocol or create a new one.

Example of parameters for the software protocol:

**Table:** 

qPCR run parameters
Step 1: 5 min at 95°C
Step 2: 15 sec at 95°C
Step 3: 30 sec at 60°C
Repeat 39 times steps 2–3
Melting: 65°C to 95°C at 0.5°C steps 5 min

  q. Press “Start Run”. The Biorad qPCR instrument will be running for approximately 2 h.
  r. If comparison between different conditions is needed, analyze gene expression using the 2^-ΔΔCt^ method.

### Protein production analysis

**Timing: 24 h**

**Timing: 24 h (for step 11)**

**Timing: 24 h (for step 12)**

This section describes how to measure protein levels either in the gels by immunofluorescence or in the supernatant via the Olink technology.

***Note:*** The overall timing corresponds to 24 h as the two techniques described below can be performed in parallel.

11. Protein localization and levels by immunofluorescence.
  ***Note:*** We selected markers expected to be robustly expressed under our experimental condition. TRPV4: a mechanosensitive calcium channel relevant to chondrocytes exposed to compressive loading; LOX, an extracellular matrix enzyme involved in collagen crosslinking and implicated in osteoarthritis; β-actin, a key cytoskeletal protein.
  a. Detach the gels from the chips and place it on a microscope slide.
  b. Fix cells by adding 100 μL of PFA 4% for 30 min at 20°C.
  c. Wash twice the hydrogels with 100 μL of PBS.
    ***Optional:*** Protocol can be paused here. Chips can be kept for 48 h at 4°C.
  d. Permeabilize cells by adding 100 μL of 0.25% Triton X-100 in PBS for 30 min at 20°C.
  e. Wash three times the hydrogels with 100 μL of PBS.
  f. Block with 100 μL of 1% BSA in PBS for 1 h at 20°C.
  g. Prepare solution with all primary antibodies in 1% BSA solution: TRPV4 Antibody [Alexa Fluor® 594] (1/150 dilution), LOX Polyclonal antibody (1/200 dilution) and β-Actin Antibody [Alexa Fluor® 647] (1/150 dilution).
  h. Incubate hydrogels with 50 μL of pool of antibodies O/N at 4°C.
  i. The day after, wash three times the hydrogels with 100 μL of PBS.
  j. Incubate the hydrogels with Alexa Fluor® 488 secondary antibody (dilution 1/500) for 1 h at 20°C, to detect LOX primary antibody.
  k. Wash the hydrogels with three times with 100 μL of PBS.
  l. Mount hydrogels with mounting medium with DAPI.
  m. Take pictures: 3 fields of 25–30 μm of depth at ×20 and 1 field at ×63 with Zeiss LSM 880 with Airyscan (confocal).
  n. Using Fiji software, convert the 3D Z-stacks images into a 2D image and measure fluorescence intensity.
    ***Note:*** By merging all signals, localization and co-localization of the proteins of interest can be studied.
12. Protein secretion in supernatants by Olink PEA.
  a. Thaw the tubes containing the cumulative medium from point 6.b.
  b. Measure protein secretion with the Olink Target 96 Inflammation panel (Olink Proteomics, Uppsala, Sweden) using 5 μL of supernatant.
  c. Analyze significantly up or down modulated proteins by generating a corresponding Vulcano-plot and NPX graph.

**Figure 2:**
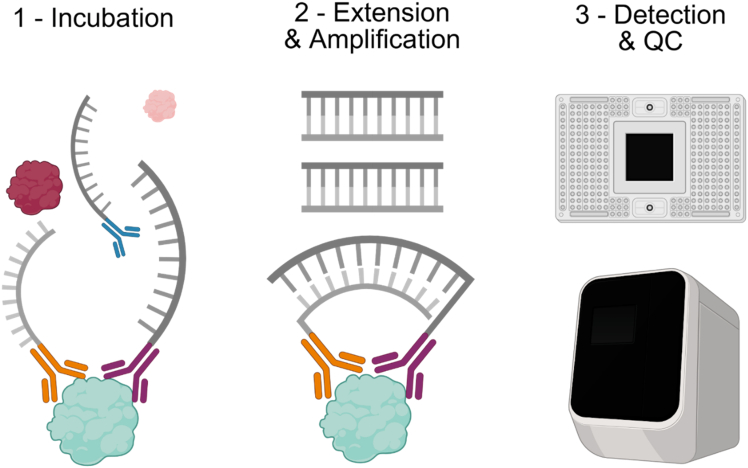
Olink Proximity Extension Assay (PEA) 1 - Antibody pairs, labelled with matching DNA oligonucleotides, bind to their respective target proteins during an 18 hours incubation and hybridize when in proximity. 2 - The resulting DNA barcodes are extended and then amplified by PCR. 3 - Amplified DNA is finally loaded onto a microfluidic chip and quantified by qPCR. After export, data QC is conducted on the NPX Signature software to get the resulting working Normalized Protein eXpression (NPX) values.

***Note:*** Olink’s PEA technology is a highly sensitivity multiplex immunoassay that enables the simultaneous quantification of 92 human inflammation-related proteins across 88 samples per plate. See Figure 2.

## Expected outcomes

Here, we present a set of complementary assays to study cartilage-on-chip systems.

**Figure 3:**
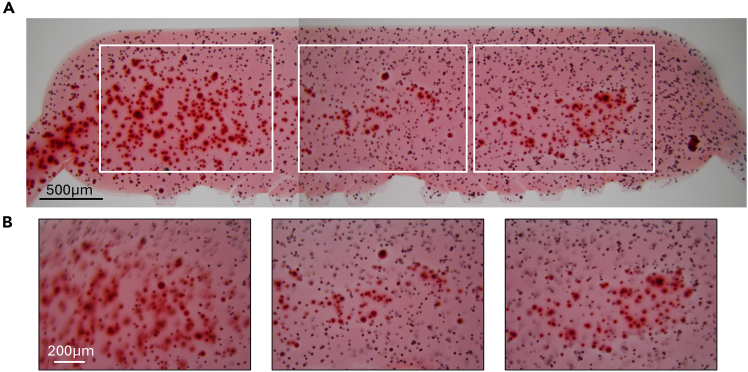
Alizarin red staining of human chondrocytes (A) Alizarin pictures of 300 mbar stimulated chips from one OA patient at ×4 magnification. (B) Alizarin pictures of 300 mbar stimulated chips from one OA patient at ×10 magnification.

Following this protocol, spontaneous cell calcification upon 300 mbar mechanical actuation can be assessed using Alizarin Red S staining. A representative image of calcified cells is shown in Figure 3 at two magnifications. For quantification, we recommend using ImageJ to calculate the area of red signal in cells.

This protocol also allows for gene expression analysis by RT-qPCR (Table 1). After RNA extraction directly from the chip, we successfully performed reverse transcription and qPCR. Ct values were obtained for both housekeeping and a cartilage-related gene (*LOX*), demonstrating the compatibility of this platform with transcriptomic analysis.

**Table 1:** Gene expression analysis

Samples	Ct values	
	GAPDH	LOX
**Replicate 1**	**28.36**	**38.07**
**Replicate 2**	**29.85**	**35.09**
**Replicate 3**	**29.07**	**34.74**

Gene expression analysis of *GAPDH* and *LOX*. Ct values extracted from the qPCR are shown in the table. The results are from 3 independent cartilage-on-chip devices that were mechanically stimulated for 1 hour a day at 1 Hz for 2 days.

**Figure 4:**
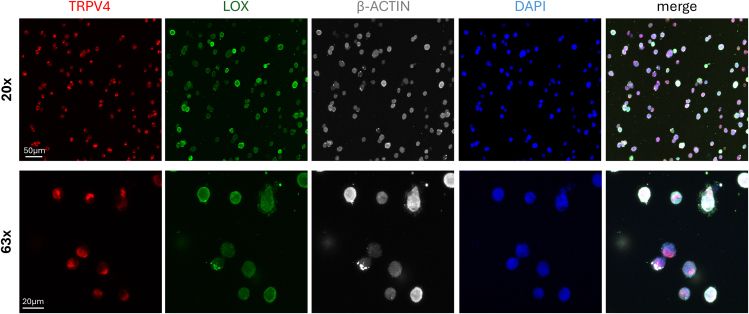
Protein expression analysis by immunofluorescence Immunofluorescences of TRPV4 (red), LOX (green), β-ACTIN (grey) with DAPI (blue) in gels stimulated at 300 mbar at 20× (upper panel) and 63× (lower panel) magnification. Images are 3D acquisitions.

In addition, immunofluorescence staining of up to four targets can be performed on-chip (Figure 4). As an example, we labeled TRPV4 (red), a mechanosensitive ion channel; LOX (green), a key enzyme involved in extracellular matrix maintenance; β-actin (grey), a cytoskeletal marker; and nuclei with DAPI (blue). Imaging was performed using confocal microscopy.

Finally, we analyzed the secretome using the Olink Target 96 Inflammation panel. All 92 inflammatory proteins passed quality control (QC), and 21 out of 92 proteins were detected above the limit of detection (LOD) which is automatically assessed for human serum, richer in proteins (Table 2). Proteins below LOD were retained in the analysis, as biologically meaningful trends may still be observed in this range while warranting further investigation and confirmation. Resulting data are expressed as Normalized Protein eXpression (NPX) values on a log2 scale, as generated by the NPX Signature software with Inter Plate Control (IPC) normalization. No additional transformations were applied prior to statistical analysis. For absolute quantification, we recommend using the Olink Target 48 panels, which provide absolute protein concentrations in pg/mL, but cover less proteins.

**Table 2:** Protein secretion analysis by Olink PEA

Above limit of detection		Below limit of detection					
Protein	NPX value	Protein	NPX value	Protein	NPX value	Protein	NPX value
4E-BP1	−0.00144	ADA	−1.24177	FGF-19	−0.9604	IL5	−0.79325
CD40	1.81111	ARTN	−0.44689	FGF-23	−0.5835	IL7	−0.33882
CD5	−0.43791	AXIN1	−0.24986	FGF-5	−0.16466	IL8	2.36873
CSF-1	2.12324	Beta-NGF	0.10395	Flt3L	−1.76144	LAP TGF-beta-1	−0.12577
CXCL6	0.5559	CASP-8	0.28559	GDNF	−0.23846	LIF-R	−0.06057
DNER	1.75798	CCL11	−0.623	HGF	−0.08925	MCP-3	0.08396
EN-RAGE	2.76873	CCL19	0.17463	IFN-gamma	1.22407	MMP-10	−0.75469
FGF-21	2.65834	CCL20	−0.73965	IL10	0.28612	NRTN	−0.66028
IL-1 alpha	0.67899	CCL23	−0.35333	IL-10RA	−0.73838	NT-3	−1.27401
IL18	0.43773	CCL25	−1.32481	IL-10RB	−0.97528	OSM	−0.20576
IL6	288429	CCL28	−0.98011	IL-12B	−1.39792	PD-L1	−1.96805
LIF	1.24949	CCL3	−0.40822	IL13	−0.18411	SCF	−0.60371
MCP-1	5.40179	CCL4	−0.57131	IL-15RA	−0.59757	SIRT2	−1.20209
MCP-2	0.53943	CD244	0.58133	IL-17A	−0.05575	SLAMF1	0.24679
MCP-4	3.21784	CD6	−1.18876	IL-17C	−0.2258	ST1A1	0.49452
MMP-1	2.60644	CD8A	−1.14746	IL-18R1	0.45926	STAMBP	−0.33884
OPG	2.27491	CDCP1	−1.11873	IL2	0.35582	TNFB	−0.52036
TGF-alpha	1.47009	CST5	−0.47145	IL-20	−0.12202	TNFRSF9	−0.35868
TNF	−0.05399	CX3CL1	−3.34784	IL-20RA	0.10968	TNFSF14	−0.08667
uPA	2.79652	CXCL1	0.69037	IL-22 RA1	0.25726	TRAIL	−0.07954
VEGFA	4.32197	CXCL10	−1.45752	IL-24	−0.35045	TRANCE	−1.7775
		CXCL11	−0.13207	IL-2RB	0.56069	TSLP	0.76994
		CXCL5	0.0435	IL33	−1.35782	TWEAK	−0.52209
		CXCL9	−0.24904	IL4	−1.56293		

Normalized Protein eXpression (NPX) values for secreted proteins in the medium of gels stimulated with 300mbar. Proteins marked as “above limit of detection” had more than 75% of samples above the assay limit of detection (assessed for human serum).

## Limitations

This protocol has several technical limitations. First, experimental users must undergo specific technical training to correctly operate the cartilage-on-chip system, including chip injection and actuation, and gel harvesting. Secondly, although the system itself requires few cells, a large initial cell quantity is required for the preparation of the gels. Finally, the analysis of secreted proteins using Olink’s PEA technology requires handling of supernatants by a certified Olink-trained scientist, or by an Olink partner laboratory.

## Troubleshooting

### Problem 1 (related to step 7)

Difficulty in capturing images of alizarin red-stained gels due to fading when washed in PBS.

### Potential solution

When processing several chips together, delay Alizarin Red staining for a few minutes so that each chip can be imaged immediately after staining, before the red color fades in PBS.

### Problem 2 (related to step 11)

Immunofluorescence staining does not work as expected.

### Potential solution

Use pre-conjugated fluorescent antibodies instead of separate primary and secondary antibodies, as this can improve signal reliability.

## Resource availability

### Lead contact

Further information and requests for resources and reagents should be directed to and will be fulfilled by the lead contact, Dr. Sonia Nasi (sonia.nasi@chuv.ch).

### Technical contact

Further information and questions about the technical specifics of performing the protocols should be directed to the technical contacts, Dr. Sonia Nasi (sonia.nasi@chuv.ch) and Dr. Carlo Alberto Paggi (carlo@chrn.co).

### Materials availability

This study did not generate any new unique reagents.

### Data and code availability

This study did not analyze/generate datasets/code.

## Acknowledgments

This study was supported by the Animal Free Research Switzerland and Proefdiervrij Netherlands to S.N. The graphical abstract was created in BioRender by D.d.H. (2026), https://BioRender.com/4rnusty and https://BioRender.com/b9rqt80.

## Author contributions

Data curation, E.F., V.C., D.d.H., and E.L.; methodology, E.F., V.C., and E.L.; writing of the original draft, E.F., D.d.H., E.L., and C.A.P.; writing – review and editing, E.F., D.d.H., C.A.P., N.B., and S.N.; conceptualization, N.B. and S.N.; supervision, N.B. and S.N.; funding acquisition, S.N.

## Declaration of interests

C.A.P. and E.L. work for chrn on-chip biotechnology B.V. and have a patent application related to this work.
